# Schizophrenia and “unmet needs”: From diagnosis to care in Italy

**DOI:** 10.1192/j.eurpsy.2019.8

**Published:** 2020-03-13

**Authors:** Silvana Galderisi, Marco A. Riva, Paolo Girardi, Mario Amore, Bernardo Carpiniello, Eugenio Aguglia, Andrea Fagiolini, Armida Mucci, Antonio Vita

**Affiliations:** 1 Department of Psychiatry, University of Campania “Luigi Vanvitelli,” Naples, Italy; 2 Department of Pharmacological and Biomolecular Sciences, University of Milan, Milan, Italy; 3 Department of Neurosciences, Mental Health and Sensory Organs, Suicide Prevention Center, Sant'Andrea Hospital, Sapienza University of Rome, Rome, Italy; 4 Department of Neuroscience, Rehabilitation, Ophthalmology, Genetics, Maternal, and Child Health (DINOGMI), Section of Psychiatry, University of Genova, Genova, Italy; 5 Department of Public Health, Clinical and Molecular Medicine—Psychiatric Unit, University of Cagliari, Cagliari, Italy; 6 Department of Clinical and Molecular Biomedicine, Psychiatry Unit, University of Catania, Catania, Italy; 7 Department of Molecular and Developmental Medicine, University of Siena, Siena, Italy; 8 Department of Clinical and Experimental Sciences, University of Brescia, Brescia, Italy; 9 Department of Mental Health, Spedali Civili Hospital, Brescia, Italy

**Keywords:** Delphi consensus, early diagnosis, schizophrenia, treatment gaps

## Abstract

**Background.:**

Schizophrenia is a leading cause of disability. People living with schizophrenia (PLWS) present unemployment, social isolation, excess mortality and morbidity, and poor quality of life. Early recognition and appropriate treatment reduce the risk of chronicity and comorbidity. Personalization and integration of pharmacological and psychosocial interventions, as well as accurate identification and management of psychiatric and somatic comorbidities, can significantly improve mental and physical health of PLWS, promoting recovery.

**Methods.:**

A three-step Delphi approach was used to explore consensus on the essential components of early recognition and intervention, personalization, and integration of care to improve schizophrenia outcome, and on barriers and challenges to close treatment gaps. The consensus involved 8 Italian experts of schizophrenia, 100 psychiatrists from academic and nonacademic settings, including representatives of Italian Society of Psychiatry, and 65 trainees in psychiatry.

**Results.:**

A strong consensus (from mostly agree to totally agree) emerged on the importance of early diagnosis (97%), standardized assessments (91%), correct management of somatic and psychiatric comorbidities (99%), and personalization and integration of care (94%). Lack of time, human resources, and training were identified as the main barriers and challenges to the translation of knowledge into clinical practice.

**Conclusions.:**

The results of this Delphi study demonstrated a strong consensus on main components of schizophrenia care, as well as on unmet needs to promote best practice and gaps between knowledge and clinical practice. The involvement of a large group of professionals and trainees in this in-depth consensus process might contribute to raise awareness and stimulate innovative strategies to improve the outcome of PLWS.

## Introduction

Schizophrenia is a mental disorder with a chronic course in up to 60% of the affected individuals and is among the top 10 causes of global disability [1,2]. Furthermore, the frequent occurrence of psychiatric and somatic comorbidities, such as depression, substance abuse (in almost 50% of patients), metabolic syndrome, cardiovascular and respiratory diseases, plays an important role in reducing person’s quality of life (QoL) and life expectancy [2].

Early diagnosis, assessment of comorbidities, as well as personalization and integration of care, may reduce the risk of chronicity and improve functional outcome [3,4]. Unmet needs of assessment and treatment, especially during the chronic phase, include negative symptoms and cognitive impairment, which predict poor functional outcome. Furthermore, psychiatric or somatic comorbidities, adherence to treatment, and integration of pharmacological and psychosocial interventions are too often overlooked in clinical practice. For all these areas, a gap between knowledge and clinical practice is acknowledged worldwide [5].

An analysis of main factors preventing early recognition and intervention (ER/EI), as well as personalization of care in chronic stages, should be carried out in National contexts to promote better standards of care for people living with schizophrenia (PLWS).

In the present article, a Delphi approach was used to share evidence-based information on schizophrenia care and assess the degree of consensus among professionals and trainees on main components of assessment and treatment to be implemented in clinical practice in Italy. The same approach was used to explore main barriers and challenges to close eventual treatment gaps.

In the following section, we report a summary of the evidence provided by the scientific board leading to concise statements on schizophrenia care.

### Early recognition and intervention

Schizophrenia research advanced our understanding of genetic and environmental risk factors, and of neurodevelopmental alterations, and holds promises for future development of “modifying” strategies aimed to change the course of the disorder, rather than merely alleviating the symptoms [4].

The identification of early symptoms, before the onset of the first psychotic episode, could interfere with the progression of the disease. Interventions on modifiable risk factors, such as social isolation or substance abuse, could prevent the conversion from the high-risk state to full-blown psychosis [3,4].

Early diagnosis and treatment, especially during the first episode of psychosis, are associated with better clinical outcome and lower rates of hospitalization, relapse, and disability [3]. However, ER/EI strategies are not fully implemented in Italy and the mean duration of untreated psychosis is still very high, as compared to the average duration in European Countries [6].

### Schizophrenia treatment unmet needs

The National Institute for Health and Care Excellence (NICE) [7] recommends psychological support for adult patients experiencing a first episode of schizophrenia or an exacerbation, and treatment with oral antipsychotics (APs). Based on a careful analysis of the risk–benefit profile, the choice of treatment should be discussed and agreed upon by psychiatrist, patient, and his/her caregiver [7].

The goals of long-term schizophrenia treatment are symptomatic and functional remission, and satisfying QoL [2,8].

The effective management of cognitive deficits and negative symptoms represent a critical unmet need of schizophrenia treatment, since these domains are closely associated with poor real-life functioning and QoL [8–11]. An increasing number of studies have highlighted the impact of negative symptoms on functional outcome [8,10,12–15]. The National Institute of Mental Health - Measurement and Treatment Research to Improve Cognition in Schizophrenia (NIMH-MATRICS) Consensus Conference on Negative Symptoms showed the difficulty in identifying appropriate measures of negative symptoms [16], and stimulated the development of new instruments whose introduction in clinical settings might improve recognition and management of this dimension. In particular, the recognition of secondary negative symptoms has notable clinical implications, as they are often amenable to treatment. For example, when these symptoms are secondary to extrapyramidal side effects (EPS) or depression, switching to a different AP or adding an antidepressant might represent effective strategies [14].

Extensive scientific literature has demonstrated the presence of cognitive deficits in all stages of schizophrenia [17–19]. The need to evaluate cognitive deficits in all PLWS, using standardized assessment tools, has gathered a broad international consensus [20], with the ultimate goal of improving schizophrenia care, by integrating AP treatment with pharmacological and nonpharmacological interventions targeting cognitive deficits and translating into improved real-life functioning and QoL.

Notwithstanding the above evidence, the assessment and treatment of negative symptoms and cognitive deficits is not carried out routinely in many academic and nonacademic mental health departments in Italy.

### Personalization of pharmacological treatment

The use of APs involves a careful assessment of the ratio between the benefits deriving from controlling the symptoms and the risk of inducing side effects. The Clinical Antipsychotic Trials of Intervention Effectiveness (CATIE) and Cost Utility of the Latest Antipsychotic drugs in Schizophrenia Study (CUtLASS) studies [21], as well as the European First-Episode Schizophrenia Trial (EUFEST) study [22], compared the risk–benefit profile of first and second generation APs (SGA), and reported that both classes present frequent side effects of comparable overall severity.

SGAs induce less EPS, but might cause more often cardio-metabolic side effects, through increase in body weight and alterations in lipid and glucose metabolism. The NICE guidelines [23] highlight that the availability of APs with different efficacy-risk profiles allows personalization of care. Clinicians should thoroughly know the benefits and adverse effects of the different APs and must evaluate the observed benefit/risk ratio for each person for any previous prescribed drug [24,25].

The NICE [24] guidelines recommend the following evaluations in the process of choosing a treatment: symptom severity, suicidal risk, agitation and aggressiveness, presence of serious cognitive deficits, and psychiatric comorbidities (e.g., substance abuse). Guidelines recommendations relevant to somatic comorbidities are reviewed in the next paragraph.

Clozapine is considered a first-line treatment for subjects with elevated suicidal risk or aggressive behavior and is superior to first-generation APs in subjects with comorbid substance abuse [26].

Despite the importance of including the evaluation of these factors in the choice of treatments, studies concerning the psychopharmacological treatment of schizophrenia not always mention these variables in the outcome evaluations [21].

Finally, the guidelines propose a stabilization phase aimed at preventing relapse, consolidating remission, and promoting the patient’s reintegration in community life [24,25].

### Somatic comorbidities and treatment of schizophrenia

Metabolic diseases such as obesity, diabetes, and dyslipidemia, and cardiovascular diseases are more frequent in PLWS than in the general population. These and other somatic comorbidities result in reduction of life expectancy of 10–20 years, compared with the general population [27–29].

Weight gain, one of the main risk factors for diabetes and cardiovascular diseases, is often overlooked in PLWS. Many factors contribute to excess weight gain, in particular those related to social context (e.g., poverty), unhealthy lifestyle (e.g., cigarette smoking and lack of physical activity), and AP side effects [29]. Furthermore, individuals with psychosis are inadequately educated and treated for these factors [30].

Despite the increased mortality for cardiovascular disease [31,32], there is a very small number of studies on these factors. In everyday clinical contexts, psychiatrists do not devote time to the screening and monitoring of these aspects (e.g., metabolic syndrome), mostly for lack of time, equipment, and training. Therefore, the integration of psychiatric services with primary care and general hospital settings is crucial [33].

The NICE guidelines [7] recommend a routine assessment of smoking, physical activity, weight, waist circumference, blood pressure, fasting blood glucose, HbA1c, and fasting lipids, to monitor the most common risk factors for cardiovascular diseases. The British Association for Psychopharmacology guidelines emphasize the need for the assessment of these risk factors before starting APs and for their monitoring over time [34]. These parameters must be re-evaluated when switching to another AP or when clinically needed. Furthermore, a routine assessment of alcohol use and smoking is suggested.

The implementation of these guidelines in clinical settings is still limited and patients do not receive adequate assessment of metabolic and cardiovascular risk.

### Subjects’ adherence to treatment

The improvement of patient’s adherence to treatment is another important aspect of schizophrenia care. It is crucial to reduce the risk of relapse and hospitalization and increase the QoL [35,36]. Understanding the factors influencing nonadherence to drug treatment is fundamental in order to identify appropriate interventions. These factors include lack of insight, patient’s attitude, risk–benefit profiles of APs, comorbidity with substance abuse, family involvement in treatment, and cognitive impairment.

Several common AP side effects (e.g., EPS, sedation, weight gain, sexual dysfunctions) might reduce adherence. Despite this, the correct management of AP side effects remains an overlooked area [37].

### Maintenance treatment, risk/benefit assessment, and antipsychotic switch

Managing maintenance treatment of PLWS is also complex due to the marked heterogeneity of individual response to treatment [38]. It is advisable to treat the patient with the AP s/he positively responded to. Undesirable side effects, such as weight gain, sedation, parkinsonism, depression, secondary negative symptoms, or sexual dysfunction, should indicate a switch to a different AP with fewer side effects [39] to reach the best balance between efficacy and undesired effects.

During the switch to a different AP, dosages should be reduced gradually to avoid a withdrawal syndrome and/or exacerbation of psychotic symptoms [38].

### Integration of pharmacological and psychosocial treatment

The complexity of schizophrenia care requires the integration of several interventions. In fact, in addition to drug therapies, it might include cognitive psychotherapy for resistant positive symptoms, cognitive remediation for cognitive impairment, interventions to improve social and work skills, and psychoeducation for patients and families [7].

The aims of integrated treatment in schizophrenia include reducing symptoms, increasing adherence to treatment, improving the continuity of care, and increasing patients’ awareness of their needs and problems, favoring autonomy and social inclusion [12,40].

A recent survey has shown that cognitive remediation [41,42] and motivational interviews have a positive impact on adherence to treatment [43]. Furthermore, available evidence suggest that cognitive remediation integrated with other psychosocial interventions (e.g., social skills training) has a positive effect on functional outcome and negative symptoms [42–44].

### Aims of the present Delphi approach

In the light of the gaps between knowledge and clinical practice summarized above, a three-step Delphi approach was used to verify the degree of consensus on: (1) essential components of early identification and intervention; (2) need for personalization of care to improve the outcome of PLWS; and (3) main barriers and challenges to close treatment gaps in Italy.

## Methods

The scientific board included eight experts of schizophrenia research and care (seven full professors of psychiatry, and one full professor of pharmacology). Survey participants included the board (except the director), a panel of 100 psychiatrists working in academic and nonacademic settings and 65 psychiatry trainees in their last 2 years of training.

Psychiatrists were selected by the scientific board according to the following criteria: at least 5 years of clinical experience, 40% among those with a leading role (Head of Department) in Mental Health Departments (MHD), 20% from academic and 20% from nonacademic settings, and 60% among staff psychiatrists from academic (30%) and nonacademic (30%) MHD. Seventeen of the 100 psychiatrists were chairpersons of the regional sections of the Italian Society of Psychiatry (SIP) and one of the scientific board members was the President of the same society.

Trainees were selected from all Italian psychiatry specialization schools among those with at least 2 years of clinical experience.

The three steps were as follows: (1) the scientific board provided a review of the evidence and discussed the topics in a first face to face focus group, and elaborated 16 statements for the web-based survey; (2) the scientific board analyzed the results and elaborated 9 in-depth questions for a second survey on barriers and challenges to the implementation of best, evidence-based care; and (3) analysis of the second survey results.

The review of the literature was carried out by the scientific board (each board member carried out a review on a topic, according to his/her main focus of interest). For each topic a narrative review was carried out and results were summarized and then discussed within the board face-to-face focus group. The narrative review included guidelines, when available, systematic reviews and/or meta-analyses, as well as consensus statements, published in English or Italian, on a specific topic.

The two surveys were organized by a professional agency, Sanitanova, with experience in the Consensus Delphi approach, in collaboration with an independent consultant psychiatrist (A.M.), expert in schizophrenia. Sanitanova is a consulting and training company in the health field, with the mission to merge the skills of clinical governance, learning and disease management through innovative models. For the design of the various solutions, Sanitanova uses consolidated internal know-how, the specialized contribution of highly qualified professionals and consultants, the supervision of a scientific board and the constant comparison with important representatives of the institutional, scientific, and academic world. The agency provided support for the preparation of the review and statements, and contributed to the analysis of the results and to drafting the manuscript.

Sanitanova sent a unique link (password-protected) to the first web survey by e-mail and respondents were asked to express their degree of agreement on each of the 16 statements by choosing a score from 1 to 5 (1 = “completely disagree”; 2 = “mostly disagree”; 3 = “somewhat agree”; 4 = “mostly agree”; 5 = “totally agree”). A consensus was reached when at least 66% of the answers were from 3 to 5.

Analysis of the first survey took place in a second face to face focus group, in which the board members prepared the second in-depth survey, aimed at highlighting barriers and challenges to the real-life implementation of the consensus statements.

The second web survey was limited to those who answered all questions in the first survey, with the exception of the trainees, who had no sufficient direct experience of the involved barriers and challenges. A unique link (password-protected) to the second survey was sent to the above respondents by Sanitanova.

The nine in-depth questions had multiple choice answers. Unlimited multiple answers were allowed for all the questions and an open answer “Other” was also allowed (for barriers not included in the multiple answers, to be specified by the respondent).

## Results

### Participants to the first survey

Respondents included 109/165 (66.1%) subjects: 6 members of the scientific board (all males), 57 psychiatrists (40 males, 17 females), including 10 SIP members and 46 trainees (14 males, 32 females). Among the participating psychiatrists, 32 were from non-Academic Mental Health Departments (15 had a leading role and 17 were staff psychiatrists), while 25 were from University Departments (8 had a leading role and 17 were staff psychiatrists).

### Results of the first survey

The results of the first survey are summarized in Table 1.

**Table 1.** Results of the first survey on early recognition, and intervention and personalization of care to improve functional outcome in schizophrenia.

Statement	Answers—n° (%)				
	Completely disagree	Mostly disagree	Somewhat agree	Mostly Agree	Totally agree
1. Mental Health Services should promote information campaigns on early diagnosis and intervention to reduce the duration of untreated active psychosis.	0 (0%)	1 (1%)	2 (2%)	14 (13%)	92 (84%)
2. An in-depth assessment of the clinical picture (including positive and negative symptoms, disorganization, depression, and cognitive deficits), and of psychosocial functioning and quality of life, through validated tools, is required.	0 (0%)	0 (0%)	10 (9%)	21 (19%)	78 (72%)
3. Taking into account clinical and anamnestic features of the individual patient, the choice of an antipsychotic drug should be based equally on the efficacy profile and on short- and long-term tolerability.	0 (0%)	0 (0%)	6 (6%)	34 (31%)	69 (63%)
4. The choice of an antipsychotic drug should consider individual factors (e.g., previous exposure to antipsychotic drugs), clinical features (e.g., suicidal risk or aggressive behavior), psychiatric comorbidity (e.g., substance use disorder), and individual preferences.	0 (0%)	0 (0%)	1 (1%)	25 (23%)	82 (76%)
5. Clozapine is the “gold standard” for treatment in patients with violent behavior or suicidal risk. In patients with comorbid substance abuse, clozapine is superior to first-generation antipsychotics.	0 (0%)	10 (9%)	26 (24%)	44 (41%)	28 (26%)
6. The choice of the antipsychotic drug must consider the tolerability profile of the single compound, according to patient’s physical health (e.g., alteration of the QT tract, cardiovascular diseases, dyslipidemia, overweight, or diabetes).	0 (0%)	0 (0%)	2 (2%)	25 (23%)	81 (75%)
7. Careful assessment of the risk factors for cardiovascular and metabolic diseases should be performed regularly. The use of standardized cardiovascular risk indicators (e.g., QRISK2) could represent a valuable tool for the clinicians.	0 (0%)	3 (3%)	12 (11%)	45 (42%)	48 (44%)
8. In the presence of health conditions and/or lifestyles at high risk for cardiovascular and/or metabolic disorders, appropriate pharmacological and nonpharmacological interventions should be provided and monitored.	0 (0%)	0 (0%)	3 (3%)	33 (31%)	72 (67%)
9. During antipsychotic drug treatment, periodic assessments of clinical parameters (BMI, abdominal circumference, arterial pressure, heart rate) and laboratory data (fasting blood glucose, glycated hemoglobin, fasting insulin, total, HDL- and LDL-cholesterol, and triglycerides) should be carried out.	0 (0%)	0 (0%)	4 (4%)	29 (27%)	75 (69%)
10. Insight level, quality of life, and compliance are strongly associated with each other, and positively correlate with adherence to pharmacological treatment.	0 (0%)	1 (1%)	16 (15%)	42 (39%)	49 (45%)
11. Psychoeducational interventions and proactivity of the Mental Health Services favor therapeutic adherence, thus improving clinical outcome, reducing stigma and health care costs, and increasing life expectancy of patients.	0 (0%)	1 (2%)	12 (11%)	34 (31%)	61 (56%)
12. A therapeutic switch should always be considered when an antipsychotic is ineffective, is effective only on some symptoms (e.g., positive), induces other symptoms (e.g., negative, cognitive) or side effects that negatively affect quality and quantity of patient’s life.	0 (0%)	0 (0%)	3 (3%)	38 (35%)	67 (62%)
13. Switching strategies depend on the clinical condition and compounds’ pharmacodynamic features.	0 (0%)	0 (0%)	14 (13%)	36 (34%)	57 (53%)
14. An in-depth evaluation of the clinical phenotype, real-life functioning and context’s features enables the identification of targeted and realistic objectives, and contribute to personalization of treatments.	0 (0%)	1 (1%)	7 (7%)	38 (36%)	60 (57%)
15. Some psychosocial interventions—such as psychoeducation, social skills training, cognitive remediation, supported-work, and cognitive-behavioral therapy for persistent psychotic symptoms—are evidence-based therapeutic approaches.	0 (0%)	1 (1%)	14 (13%)	31 (30%)	58 (56%)
16. People living with schizophrenia must receive from Mental Health Services integrated treatments, including well-tolerated and effective pharmacotherapy, evidence-based psychosocial interventions and physical health monitoring. The aim of integration is to promote person’s recovery and quality of life.	0 (0%)	0 (0%)	2 (2%)	19 (18%)	83 (80%)

A consensus was reached on all statements of the first survey, although for some of them the degree of agreement was lower than for others (the percentages of “totally agree” varied from 28 to 78%) and on a few statements there were small percentages of disagreement (from 2 to 10%).

A strong consensus (from mostly agree to totally agree) emerged on the importance of early diagnosis (97%), standardized assessments (91%), correct management of somatic and psychiatric comorbidities (99%), and personalization and integration of care (94%).

Disagreement was expressed on clozapine treatment in case of aggressive or suicidal risk or substance abuse, and on monitoring of cardiovascular and metabolic risk factors.

Supplemental materials include a detailed description of agreement percentages for category of participants.

### Participants to the second survey

Sixty-three of the 109 respondents to the first survey were contacted for the second survey, including the 6 Scientific Board members, as well as the 57 psychiatrists. Respondents to the second survey included 52/63 (82.5%) of those who were contacted, 39 males and 13 females.

### Results of the second survey

The results of the second survey are summarized in Table 2 and a detailed description of the survey results across categories is available in the Supporting Information.

**Table 2.** Results of the survey on barriers and challenges to improve schizophrenia care.

Question	Answers	*N* (%)
1. The average duration of active psychosis in Italy is about 50 months. What are the main barriers to the implementation of early recognition and intervention strategies?	Lack of human resources	39 (35%)
	Lack of economic resources	26 (23%)
	Fear of stigma	18 (16%)
	Lack of training	25 (22%)
	Other	5 (4%)
2. What are the main obstacles to the routine use of validated tools for clinical assessment?	Reduced availability of validated tools	7 (6%)
	Lack of training in the use of these tools	41 (37%)
	Lack of time	25 (23%)
	Lack of professionals (e.g., psychologists)	34 (31%)
	Poor utility in clinical practice of the standardized assessment of positive symptoms, disorganization, negative symptoms, depression, cognitive deficits, psychosocial functioning, and quality of life	2 (2%)
	Other	1 (1%)
3. When the choice of an antipsychotic drug is not based equally on the efficacy profile and on short- and long-term tolerability, and does not consider individual factors (e.g., previous exposure to antipsychotic drugs), clinical features (e.g., suicidal risk or aggressive behavior), psychiatric comorbidity (e.g., substance use disorder), and individual preferences, what are the main reasons?	Reduced availability of antipsychotic drugs in the service/department	17 (14%)
	Limited information on pharmacodynamic profile of antipsychotic drugs	18 (15%)
	Limited information on differences in efficacy among antipsychotic drugs	15 (12%)
	Limited information on tolerability profile of antipsychotic drugs	10 (8%)
	Poor experience with the use of some antipsychotics in clinical practice	16 (13%)
	Costs of drugs	18 (15%)
	Greater emphasis on the effectiveness profile in the choice of short- and long-term treatment	19 (15%)
	Greater emphasis on the tolerability profile in the choice of short- and long-term treatment	10 (8%)
	Other	0 (0%)
4. Why clozapine is not used in case of suicidal risk or aggressive behavior and/or comorbid substance abuse?	Patient’s resistance to carry out weekly blood count	34 (34%)
	Cardiovascular risk in patients with comorbid substance abuse	8 (8%)
	Metabolic risk	16 (16%)
	Excessive sedation	12 (12%)
	Hypersalivation	10 (10%)
	Epileptogenic risk	3 (3%)
	Other	17 (17%)
5. What are the main obstacles to routine assessment of cardiovascular and metabolic risk factors?	Lack of human resources	17 (19%)
	Lack of economic resources	4 (5%)
	Limited information on the use of standardized indicators	38 (43%)
	Lack of linkage with specialized structures	27 (31%)
	Other	2 (2%)
6. What are the main obstacles to the implementation of interventions for patients with somatic comorbidity?	Poor acceptance of these interventions by patients	21 (20%)
	Lack of linkage with specialized structures	30 (28%)
	Limited information about possible interventions	27 (26%)
	Lack of human resources	17 (16%)
	Lack of economic resources	7 (7%)
	Other	3 (3%)
7. What are the main obstacles to implementation of psychoeducational interventions?	Lack of human resources	41 (42%)
	Lack of economic resources	13 (14%)
	Lack of training	42 (43%)
	Other	1 (1%)
8. What are the main obstacles to AP switch if necessary?	Fear of symptomatic worsening during the switch	31 (26%)
	Reduced confidence in the advantage of the switch, in terms of clinical response	26 (22%)
	Lack of training on correct switching methodology	17 (14%)
	Difficulty in finding an alternative drug due to the comparable efficacy of antipsychotic drugs	1 (1%)
	Conviction of the overlapping of antipsychotic drugs in terms of effectiveness	20 (17%)
	Lack of time or difficulty to carry out an adequate monitoring after switching	11 (9%)
	Resistance of patient and his family with respect to the switch	12 (10%)
	Other	1 (1%)
9. What are the main barriers to implementation of evidence-based psychosocial interventions in clinical practice?	Lack of human resources	40 (30%)
	Lack of economic resources	18 (14%)
	Lack of adequate structures	19 (14%)
	Lack of opportunities (e.g., for supported work)	20 (15%)
	Lack of training	35 (26%)
	Other	1 (1%)

In the second survey unlimited multiple answers were allowed for all the questions and an open answer “Other” was also allowed. The percentages were calculated on the total number of answers for each question.

The results clearly indicated that treatment gaps are diffusely acknowledged by professionals in Italy. As hypothesized by the board, main barriers and challenges were lack of time, human and financial resources, and training. In particular, these barriers were indicated as the most relevant for the poor implementation of ER/EI campaigns and services in Italy. The same barriers were also indicated for the suboptimal utilization of standardized tools for assessment of psychopathology, cognitive deficits, psychosocial functioning, and quality of life in PLWS. Lack of human resources and training were the main barriers to the implementation of psychoeducational and other evidence-based interventions.

For somatic comorbidities, as well as cardiovascular and metabolic risk management, the lack of information on standardized tools and the lack of liaison with specialized tertiary-care units emerged as the most important challenges and barriers. Another factor emerging for the gap in management of somatic comorbidities was the patient poor adherence to treatment targeting these comorbidities.

For management of AP treatment, including switch to another AP in case of poor response or side effects, no clear pattern emerged, and all factors suggested by the board were equally indicated as important determinants of treatment gaps.

## Discussion

A three-step Delphi approach was used for the first time in Italy to share evidence-based information on schizophrenia care, assess the degree of consensus among professionals and trainees on main components of assessment and treatment to be implemented in clinical practice, and evaluate main barriers and challenges to close eventual treatment gaps.

The results of the first survey revealed a large consensus among experts, professionals, and trainees in psychiatry on all investigated aspects of schizophrenia care.

In particular, the importance of ER/EI to reduce the duration of untreated psychosis had a very large consensus indicating that the Italian psychiatric community is aware of the need to diffusely increase ER/EI programs. However, although guidelines for the implementation of ER/EI are available since 2007 in Italy (http://www.salute.gov.it/imgs/C_17_pubblicazioni_714_allegato.pdf), and ER/EI programs were implemented in several regions, national ER/EI programs are still missing. Our results indicate that main obstacles include lack of human and economic resources and training, although several studies demonstrated a net saving in direct health-related and indirect costs with ER/EI services versus standard care (http://www.dh.gov.uk/en/Publication). A nation-wide implementation of these services would free both financial resources, that could be reinvested in training, and human resources.

The survey also demonstrated a large consensus on the need for careful assessment, using validated tools, of positive and negative symptoms, disorganization, depression, and cognitive impairment, as well as psychosocial functioning and QoL, to guide personalization of care. The Italian Network for Research on Psychoses introduced validated and standardized psychopathology assessment tools [8,10,18,45–48] as well as state-of-the-art instruments to assess cognitive deficits and psychosocial functioning in schizophrenia. However, the use of these tools is not largely diffused in the clinical practice, due to lack of information, training, and time. The involvement of scientific and professional organizations in the promotion and dissemination of these instruments, might contribute to close this gap.

The opportunity to equally weigh efficacy and side effects in the choice of an AP drug for each individual patient also received a large consensus as did the need for treatment aimed to improve schizophrenia, beyond the reduction of psychotic symptoms. In fact, a large consensus was expressed for the need of considering a switch when only some symptoms are ameliorated or other emerged during treatment (e.g., negative or depressive symptoms), or when the prescribed AP poses a considerable cardiovascular or metabolic risk in vulnerable subjects.

A further important area of consensus involved patients’ adherence to treatment. The improvement of patient’s adherence to treatment is a key aspect of schizophrenia care to reduce the risk of relapse and hospitalization, and increase real-life functioning and QoL [35,36]. AP lack of efficacy or common side effects (e.g., EPS, sedation, weight gain, sexual dysfunction) are possible causes of poor adherence.

There was a high level of agreement on the need to switch AP in case of persistent symptoms or side effects. However, several obstacles might limit the switch in the clinical practice; in particular, poor information on efficacy/tolerability profiles of APs and unbalanced attitudes to emphasize efficacy or tolerability were indicated as main obstacles by the second survey. Most of these obstacles can be removed through national awareness campaigns on AP efficacy and side effect profiles, or regular organization of CME courses for psychiatrists, general practitioners, and trainees in psychiatry, providing training on AP comparative efficacy and tolerability.

A small percentage of disagreement (10%) was expressed on the use of clozapine in case of suicide risk, aggressive behavior, or comorbid substance use. An even smaller percentage of disagreement (6%) was also expressed on the need for cardiovascular risk monitoring, using standardized indicators. In both cases, the large majority of respondents agreed with the proposed statement, and the second survey indicated a gap between knowledge and clinical practice.

In the case of clozapine treatment in case of suicidal risk, violence or substance use, the main reasons for the gap are concerns regarding its adverse side effects, particularly metabolic and cardiovascular risk, and excessive sedation, as well as poor compliance of patients with blood count monitoring for the risk of agranulocytosis. For cardiovascular and metabolic risk monitoring, the lack of connections between specialist facilities and of human resources were indicated as the main obstacles to implementation in clinical practice. When there are cardiovascular and/or metabolic diseases, appropriate pharmacological and nonpharmacological interventions should be implemented and monitored, but often there is little attention to the problem by the patient and a lack of trained staff among psychiatrists.

Another important area showing a gap between knowledge and clinical practice emerged from our results: the need for correct management of somatic comorbidities, and of cardiovascular and metabolic risk factors. The lack of information on standardized tools for a thorough assessment of somatic comorbidities and risk factors, and poor liaison with specialized tertiary-care units emerged as the most important challenges to close the gap. Patients’ poor adherence to treatment of these comorbidities and strategies addressing risk factors (e.g., pharmacologic treatment of diabetes) also emerged as a reason for the poor management of somatic comorbidities.

A Delphi consensus on physical health management in people with severe mental illness recommended an increased cooperation among health professionals (e.g., general practitioners, cardiologists, and psychiatrists) [49].

The implementation in Mental Health Departments of evidence-based integrated and personalized treatment plans, shared with PLWS and their unofficial caregivers, are important steps to promote adherence to treatment, improve clinical outcome, reduce stigma and health care costs, and increase patient life expectancy. The use of innovative strategies, such as the implementation of web-based versions of psychosocial interventions (e.g., psychoeducation), might be helpful to overcome the lack of human resources and training, identified as important barriers to the implementation of psychosocial interventions. Awareness campaigns addressing the urgent need for integrated and personalized treatments, including effective and well-tolerated pharmacotherapy, and physical health monitoring, may further improve the current clinical practice. The ultimate goal is to promote patient’s recovery and improve her/his QoL. Policy makers should be aware that pursuing this target will reduce direct and indirect costs associated with chronicity, hospitalization, psychiatric and somatic comorbidities, and consequent disability.

## Conclusion

The results of this consensus Delphi approach included a high level of agreement among the board of experts, a group of Italian psychiatrists and a group of trainees in psychiatry on the need to increase the number of services for ER/EI for psychotic disorders in Italy, as well as to improve models of care in schizophrenia by promoting the implementation of integrated and personalized treatment plans.

The results clearly indicated that gaps between knowledge and clinical practice are diffusely acknowledged by Italian professionals and are related to lack of time, human and financial resources, as well as of adequate training.

These results highlight the need for coordinated action of professionals and other stakeholders to overcome existing barriers and ultimately improve the functional outcome of PLWS in Italy.

This paper, resulting from the involvement of a large panel of professionals, including representatives of the largest organization of psychiatrists and of trainees, might stimulate further discussion of the addressed topics and contribute to innovative actions aimed to improve the outcome of PLWS.

## Study Limitations

Some limitations of the study should be acknowledged. In particular, we did not address the efficacy of other APs, apart from clozapine, in patients with violent behavior and in subjects with substance use disorders. In addition, the consensus relevant to AP switching strategies failed to include the role of pharmacokinetic properties of APs (such as long-acting formulations). It should also be acknowledged that current findings, while reflecting different settings of mental health care provision in Italy, might not generalize to other Countries and do not cover the role and views of all stakeholders in the field of mental health (e.g., social workers, primary care doctors, organizations of patients, and relatives).
